# Development of computable guidelines: GIN McMaster guideline development checklist extension for computable guidelines

**DOI:** 10.1002/gin2.70023

**Published:** 2025-05-02

**Authors:** Chirine Chehab, Stacy Lathrop, Christopher G. Harrod, James Kariuki, Derek Ritz, Maria Michaels

**Affiliations:** ^1^ Centers for Disease Control and Prevention (CDC) Atlanta Georgia USA; ^2^ National Center for Biotechnology Information, National Library of Medicine (NLM) National Institutes of Health (NIH) Bethesda Maryland USA; ^3^ Society of Critical Care Medicine (SCCM) Los Angeles California USA; ^4^ ecGroup Inc Ancaster Ontario Canada; ^5^ Guidelines International Network North America Region (GIN‐NA) Perthshire Scotland

**Keywords:** clinical guidelines, computable guidelines, GIN‐McMaster guideline development checklist, guideline developers, informaticists

## Abstract

**Background:**

Transforming Clinical Practice Guideline (CPG) recommendations into computer readable language is a complex and ongoing process that requires significant resources, including time, expertise, and funds. The objective is to provide an extension of the widely used GIN‐McMaster Guideline Development Checklist (GDC) and Tool for the development of computable guidelines (CGs).

**Methods:**

Based on an outcome from the Human Centered Design (HCD) workshop hosted by the Guidelines International Network North America (GIN‐NA), a team was formed to develop the checklist extension. The team included guideline developers, researchers, implementers, and informaticists who reviewed the GDC and developed a list of additional requirements to help guideline developers author clearer, more implementable narrative guideline recommendations (referred to as knowledge level 1, or L1 recommendations) and ensure conformance‐testable attributes of the different artifacts of clinical guideline recommendations. The team vetted this list with guideline development organizations and health informatics experts to validate it, for clarity, usability, and effectiveness. The team used an iterative process to determine the final extension components for CG development guidance.

**Results:**

The team identified nine components that complement the topics included in GDC for developing, implementing, and adopting CG recommendations.

**Conclusion:**

This study demonstrates that the defined principles in the L1 Checklist, grounded in current guideline development standards, may significantly enhance the writing, development, and implementation of computable recommendations. Collaboration among guideline developers, implementers, and informaticists from the outset is crucial for achieving effective integration of these guidelines into clinical workflows. Future work should focus on assessing this extension within various ongoing learning initiatives and point‐of‐care digitization efforts, including the scholarly communications ecosystem and learning health systems, to further improve healthcare delivery.

## Highlights

- This is an extension to the GIN McMaster Guideline Development Checklist, a foundational instrument to evaluate and enhance the readiness of narrative clinical guidelines for digitization, addressing a critical gap in the pathway to computable guideline development.
- The development of the checklist is anchored in evidence and shaped by iterative feedback from a multidisciplinary network of global stakeholders, ensuring its methodological integrity, relevance, and practical utility across diverse healthcare contexts.
- The extension checklist represents a strategic advance in bridging traditional guideline formats and the requirements for electronic health record integration, thereby promoting the seamless translation of evidence into practice.
- The extension checklist is scalable and flexible, applicable to a diverse range of guidelines, with the goal of establishing it as a broadly used tool for supporting consistency and quality in future guideline digital transformation efforts.

## 1 BACKGROUND

Clinical Practice Guidelines (CPGs) present systematically developed recommendations intended to assist healthcare organizations, clinical providers, patients, and public health agencies in making decisions about optimized care and public health. Over the past decade, numerous initiatives have provided guidance on the development and implementation of CPGs for diverse users of these guidelines.^1^, ^2^ In 2014, the Guidelines International Network (GIN) and McMaster University collaboratively developed the GIN‐McMaster Guideline Development Checklist (GDC).^3^ The GDC supports the development of methodologically rigorous, transparent, and evidence‐based written CPGs and serves as a valuable tool for evaluating and improving the quality of these guidelines.^3^ The GDC includes 18 general topics focused on enhancing the overall quality and reliability of clinical guidelines; these topics allow for customization based on the user's specific area of interest or relevance.^3^ The GDC is complemented with three recent extensions: (1) for rapid guidelines development that addresses public health emergencies, (2) for the integration of quality improvement and quality assurance in guidelines, (3) for health equity.^4^, ^5^, ^6^, ^7^, ^8^

Concerns have been raised by guideline development experts related to inconsistencies in guideline methodology, content, and evidence‐based recommendations. This can create challenges incorporating guidelines in ongoing digitization initiatives within both the scholarly communications ecosystem and the learning health system (LHS).^9^, ^10^ The scholarly communications ecosystem refers to the interconnected network of processes, entities, and technologies involved in the creation, dissemination, and utilization of scholarly knowledge and research; it encompasses various components and customers within academia and the wider community.^9^ The LHS emphasizes the integration of data, knowledge, and practice to support policy and planning, public health, and personalization of care.^10^, ^11^ Accordingly, an initiative on Adapting Clinical Guidelines for the Digital Age (ACG), led by the Centers for Disease Control and Prevention (CDC), began in 2018 with the aim to redesign and improve written and computable guideline (CG) development, implementation, and standardization for more actionable and integrated guidance at the point‐of‐care.^12^

The ACG initiative incorporated multidisciplinary perspectives following an adapted Kaizen approach to improve multi‐organizational, multidisciplinary complex processes.^13^ The initiative focused on five areas: guideline creation, informatics, implementation, communication, and evaluation. The ACG workgroups delivered standards, processes, and tools that included a Fast Healthcare Interoperability Resources (FHIR®)‐based technical standard for CGs known as FHIR® Clinical Guidelines or CPG‐on‐FHIR®, a 12‐phase integrated process and model for co‐development and implementation of written and computable guidelines, and an evaluation framework for the integrated process.^8^, ^14^, ^15^ Computable guidelines are “the representation of written guideline recommendations in a computer‐interpretable format”.^8^, ^12^, ^13^, ^15^ The CPG‐on‐FHIR® implementation guide (IG) provides a means of creating a computable representation of narrative clinical guideline recommendations that is faithful to guideline intent and supports the derivation of downstream capabilities such as cognitive and decision support, quality measures, case reports, and documentation templates that direct clinical documentation in support of determining guideline compliance.^14^ As of the summer of 2023, the CPG‐on‐FHIR® IG included three checklists associated with three knowledge representation levels: semi‐structured (L2), structured (L3), and executable (L4).^14^, ^16^, ^17^

Building on the ACG effort, GIN North America (GIN‐NA) launched a Human‐Centered Design (HCD) workshop on “Bringing Guidelines to the Digital Age.” HCD is an iterative creative approach that emphasizes the needs and desires of user and prioritizes stakeholder engagement and feedback throughout the process.^18^ Participants worked in teams during and after the workshop to design solutions that address key pain points related to guideline development and implementation.^18^ One of these teams focused on Standard Operating Procedures (SOPs) to help guideline developers make narrative guideline recommendations clearer and easier to implement. The SOP team iteratively developed a checklist to include a structured template for developing, wording, and structuring narrative recommendations and their pertinent metadata that can be represented in a computable format.

This article reports on GIN‐NA's HCD effort to develop an extension of the widely used GDC for the adoption and implementation of CGs. This checklist extension is intended to be used as a tool to help guideline developers produce narrative clinical guidelines, knowledge level 1 (L1), in clearer, more consistent, and more structured ways. As a result, key information like metadata, evidence, and recommendations may be consistently and successfully implemented by informaticists or implementers in computable formats for use in various digital tools, such as clinical decision support systems or clinical quality measures. Such systems can improve patient outcomes through evidence‐based medical practice in a LHS based on Findable, Accessible, Interoperable, and Reusable (FAIR) principles, therefore creating a continuous learning cycle of evidence generation, translation to practice, and practice‐driven data collection and use.^19^ The scope may include clinical practice guidelines and their related systematic reviews, evidence reports, and supplementary data and materials.

## 2 METHODS

Following the HCD creative approach for problem solving, the GIN‐NA SOP team included guideline developers, researchers, implementers, and informaticists. The SOP team started with the identification of elements needed for developing computable guideline recommendations within the 18 topics of the current GDC guideline development process (Table 1). These elements were initially broad recommendations related to the inclusion of informaticists, structured guideline wording, computable representations, and implementation considerations. To ensure these elements align with standards, the team adopted standards used by several guidelines authorities including the U.S. Institute of Medicine (IoM) Clinical Practice Guidelines We Can Trust, CPG‐on‐FHIR®, the World Health Organization (WHO) SMART Guidelines, and the U.S. National Center for Biotechnology Information (NCBI) Bookshelf Tagging Guidelines.^2^, ^3^, ^14^, ^24^ The team also considered the possibility of building translations between the American National Standards Institute/National Information Standards Organization (ANSI/NISO) and the Journal Article Tag Suite (JATS) standard used by publishers and archives to exchange full‐text narrative CPGs published in journals.^25^ In addition, the team looked at the derivative Book Interchange Tag Suite (BITS) used by the U.S. National Library of Medicine to normalize, archive, display, and index CPGs developed by the WHO and U.K. National Institute for Health and Care Excellence, and CPG‐on‐FHIR® formats.^2^, ^14^, ^24^

**Table 1:** Identified elements for CGs in the GDC topics.

GDC topic	Elements identified for computable guidelines (CGs)
1. Organization, budget, planning and training	It is recommended to map the IT context of intended CG users as part of the infrastructure.
2. Priority setting	No distinct elements identified for CGs
3. Guideline group membership	It is recommended that informaticists are included in the guideline group. It would be useful for every member of the guideline authoring team to have at least a rudimentary understanding of informatics concepts used to develop CG recommendations. A simple training module may be sufficient. If an authoring tool is to be used, each member should understand how to use it.
4. Establishing guideline group processes	No distinct elements identified for CGs
5. Identifying target audience and topic selection	No distinct elements identified for CGs
6. Consumer and stakeholder involvement	In addition to including informaticists in the guideline group, it would be important to also have informaticists and implementers co‐developing and testing computable artifacts (such as algorithms, value sets that define data elements, etc.) in parallel with the recommendation development. Iteration between the draft computable artifacts and the draft narrative recommendations could help make the narrative version clearer and more actionable and the computable version more effective to the recommendations' intent for optimal patient care.
7. Conflict of interest considerations	No distinct elements identified for CGs
8. Patient/population, intervention, comparison, and outcomes (PICO) question generation	The applicable population, intervention, comparator, and outcomes of the PICO question should be expressed using specific data elements and value sets from standardized data sets, such as the International Patient Summary (IPS), United States Core Data for Interoperability (USCDI), or U.S. National Institutes of Health (NIH) Common Data Elements.^20^, ^21^, ^22^
9. Considering importance of outcomes and interventions, values, preferences and utilities	No distinct elements identified for CGs
10. Deciding what evidence to include and searching for evidence	No distinct elements identified for CGs
11. Summarizing evidence and considering additional information	No distinct elements identified for CGs
12. Judging quality, strength or certainty of a body of evidence	No distinct elements identified for CGs
13. Developing recommendations and determining their strength	All recommendations must be expressed in an unambiguous format, including traceability to the source evidence.
14. Wording of recommendations and of considerations of implementation, feasibility and equity	All recommendations must be expressed with unambiguous language using Conditions, Actions, Resultant Data, and State (CARDS) methodology (i.e. when these conditions are clearly observed, these actions are recommended, and the outcome of taking these actions should be these resultant data and state).^23^ Each element of the CARDS methodology should be expressed using a relevant data model.
15. Reporting and peer review	Review of the resulting narrative and computable guidelines by an informaticist is essential to ensure guideline recommendations are implementable.
16. Dissemination and implementation	Easy‐to‐use digital tooling should be employed at every phase of CPG development so that the process of publishing the CG is not a disconnected afterthought but rather an inherent outcome of the authoring process. It would be useful to adapt existing authoring and publishing tools and standards to support translation to FHIR® standards for implementation into healthcare systems and infrastructure.
17. Evaluation and use	It will be essential to integrate technology and data analytics in the guideline's evaluation, including leveraging observational data within Electronic Health Records (EHRs) to assess the real‐world effectiveness of recommendations using advanced data analysis tools and conformance‐testing of computable artifacts. Closing the loop by making analytics on evaluation and use of recommendations available to the authoring guideline developers will provide useful implementation insights that are not as easily gleaned from systematic reviews to incorporate into guideline updates.
18. Updating	It is recommended to use versioning algorithms and tools to ensure timely update of the guideline recommendations and version comparisons. Versions should be mapped between narrative and computable formats to ensure fidelity to the latest evidence supporting guidance.

**Figure 1:**
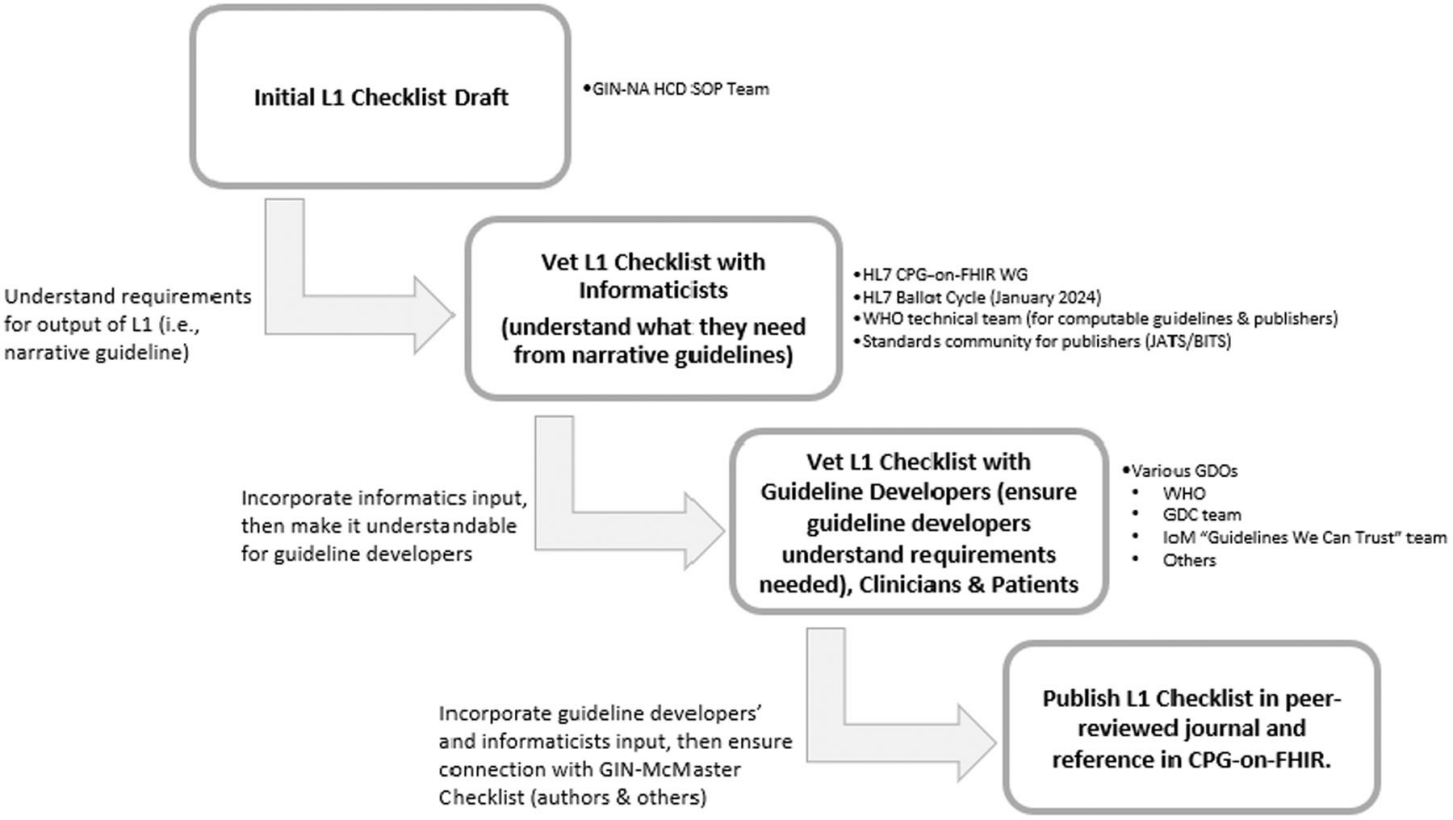
L1 Checklist development and vetting process.

The SOP team then developed a draft list – the “L1 Checklist” – of crucial approaches to help guideline developers author clearer, more actionable narrative guidelines (L1) by defining testable attributes of different clinical guideline recommendations artifacts. The draft checklist was systematically evaluated through multiple validation steps to assess its clarity, usability, and effectiveness in supporting computable guideline (CG) development. To ensure clarity, the team vetted this list with the guideline development organizations and informatics experts, totaling 25 individuals, to determine whether each checklist item improved the precision and consistent interoperability of L1 recommendations. Structured discussions, written feedback, and expert consensus methods (e.g., Delphi rounds) were used to refine ambiguous or redundant elements. (Figure 1). To assess effectiveness, the team published a preprint of the first draft of the L1 checklist^26^ to invite broader input from the guideline development and health informatics communities. The preprint was also included in the HL7® CPG‐on‐FHIR® ballot for the January 2024 cycle, allowing for international review by health IT standards experts.^27^ The team systematically analyzed comments from these reviewers to determine whether the checklist facilitated more structured, computable, and testable guideline recommendations. For usability, the team shared the draft L1 checklist with the editors of the HL7® CPG‐on‐FHIR® implementation guide and the WHO SMART guidelines team to assess its practicality. Stakeholder feedback based on the discussions that included perspectives of guideline developers, informaticists, and other interested parties was mapped to specific checklist items to ensure iterative improvements. The iterative refinement process involved consolidating feedback from multiple sources, tracking revisions, and reaching consensus on modifications. All suggested changes, discussions, and rationale for adjustments were documented to ensure transparency and reproducibility in developing the final checklist.

## 3 RESULTS

Following the vetting process, the team received feedback on the draft L1 Checklist (i.e., preprint) and no comments specifically related to the L1 checklist from the CPG‐on‐FHIR® ballot.^26^ The feedback captured the nuanced perspectives of the different customers of guideline development and implementation. Overall, the feedback emphasized the significance of developing a comprehensive and topic‐specific L1 checklist. The nine components of the L1 Checklist were deemed essential to CPG development and implementation in computable format. Participants mentioned that the L1 checklist offered a highly needed approach to streamline the existing process, employing more structured and consistent narrative language to guide the main contributors: guideline developers, informaticists, and implementers. The feedback highlighted gaps in the GDC with respect to CGs and strengthened the nine components outlined in the draft L1 checklist, placing particular focus on the guideline structure, documentation, and updates components. In response, the SOP team addressed these gaps to enhance the CGs development process. The refined final L1 checklist indicates whether the topic is applicable to guideline developers or informaticists and implementers or both. It also includes links to useful resources with suggested methodology for applying its components (Supporting Information: Appendix 1).

**L1 Checklist Items**

**Item 1‐Policy**

(Applicable to Guideline Developers)

(New item linked to GDC topic 1 “Organization, Budget, Planning and Training”)

Provide a clear policy and link with a persistent identifier to public documentation of procedures used by the organization for guideline recommendations development, including how it establishes transparency, composes its guideline development group, manages conflicts of interest, reviews and rates evidence using rigorous methods, conducts peer review, updates the guideline versions, manages data, and distributes/publishes guidelines recommendations.

**Item 2‐Scope**

(Applicable to Guideline Developers)

(Extended item linked to GDC topic 5 “Identifying Target Audience and Topic Selection” and topic 8 “Patient/population, Intervention, Comparison, and Outcomes (PICO) Question Generation”)

Clearly define the scope of the guideline development project, including who the guidelines are for (e.g., subject matter experts (SMEs), providers, patients, etc.), what clinical domains they would cover, and what settings and contexts they may be practiced at.

**Item 3‐Goal(s)**

(Applicable to Guideline Developers)

(Extended item linked to GDC topic 2 “Priority Setting”)

Clearly articulate why a guideline development project is needed.

**Item 4‐Contributor(s)**

(Applicable to both Guideline Developers and Informaticists/Implementers)

(Extended item linked to GDC topic 3 “Guideline Group Membership”)

Ensure the involvement of informaticists and implementers in the guideline development panel at the start of the process to help identify areas in the guideline that require clarification and translation into CG recommendations using appropriate tools, data elements, and value sets.

**Item 5‐Knowledge Levels Requirements**

(Applicable to Informaticists/Implementers)

(New item to GDC topics)

Provide clear and tangible requirements of the semi‐structured knowledge level 2 (L2), structured knowledge level 3 (L3), and executable knowledge level 4 (L4) so that guideline developers can write narrative (L1) recommendations that are readily translated by the informaticists into the other knowledge levels.^28^ These levels represent a structured progression from traditional narrative text to fully computable formats in CPG development.

**Item 6‐Guideline Question formulation**

(Applicable to Guideline Developers)

(Extended item linked to GDC topic 8 “Patient/population, Intervention, Comparison, and Outcomes (PICO) Question Generation”)

**Figure 2:**
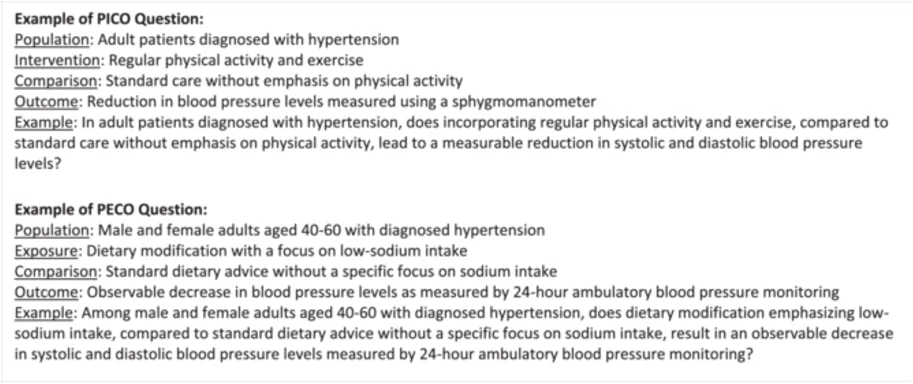
Examples of PICO and PECO questions related to the management of hypertension that are measurable and/or observable.

Formulate measurable and observable clinical questions using the Patient/Population, Intervention, Comparison, and Outcomes (PICO) or Patient/Population, Exposure, Comparison, and Outcomes (PECO) approaches.^2^ The PICO/PECO question components should be expressed using specific data elements and value sets from standardized data sets, such as IPS, USCDI, or NIH Common Data Elements^20^, ^21^, ^22^; Figure 2 shows examples of clinical questions following these approaches.

**Item 7‐Guideline Structure**

(Applicable to both Guideline Developers and Informaticists/Implementers)

(New item linked to GDC topic 12 “Judging Quality, Strength or Certainty of a Body of Evidence”)

**Figure 3:**
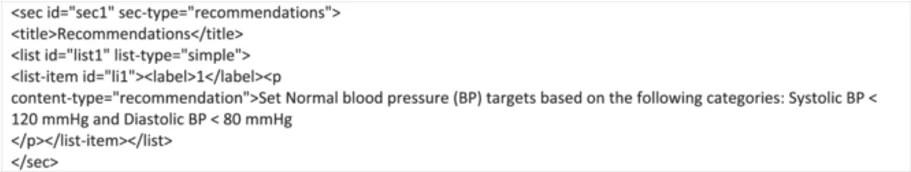
Example of a JATS structured format of a recommendation for the management of hypertension.

**Figure 4:**
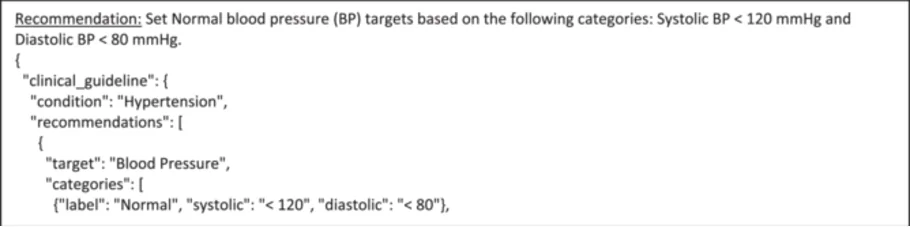
Example of a FHIR machine‐readable format of a recommendation for the management of hypertension.

Use a valid grading system and approach for CG recommendations, such as the Grading of Recommendations Assessment, Development and Evaluation (GRADE) methodology.^29^ CG recommendations need to be written and styled in an identifiable, consistent format (i.e., contained in a labelled recommendation section with each recommendation labelled separately so it may be assigned a unique identifier) so that they may be encoded and extracted from narrative contexts for translation across interchangeable formats (e.g., conditional statements using “If… then”) including the strength of the recommendation (e.g., strong, conditional). Examples are shown in Figures 3 and 4.

**Item 8‐Documentation requirements per recommendation**

(Applicable to both Guideline Developers and Informaticists/Implementers)

(New item linked to GDC topic 14 “Wording of Recommendations and of Considerations of Implementation, Feasibility and Equity” and topic 16 “Dissemination and Implementation”)

Utilize existing applications, consistent templates, or authoring and conversion tools that facilitate the extraction of individual recommendation statements to be translated for execution. Such translations may be specified algorithms or decision trees or other computable representations that permit guidelines to be successfully integrated and implemented, allowing for systematic and consistent decision‐making based on specific conditions or criteria. These resources can streamline the guideline development process and ensure a standardized approach to their documentation. Articulate all possible care options explicitly, incorporating known variables and contextual factors necessary for optimal decision‐making.

**Figure 5:**
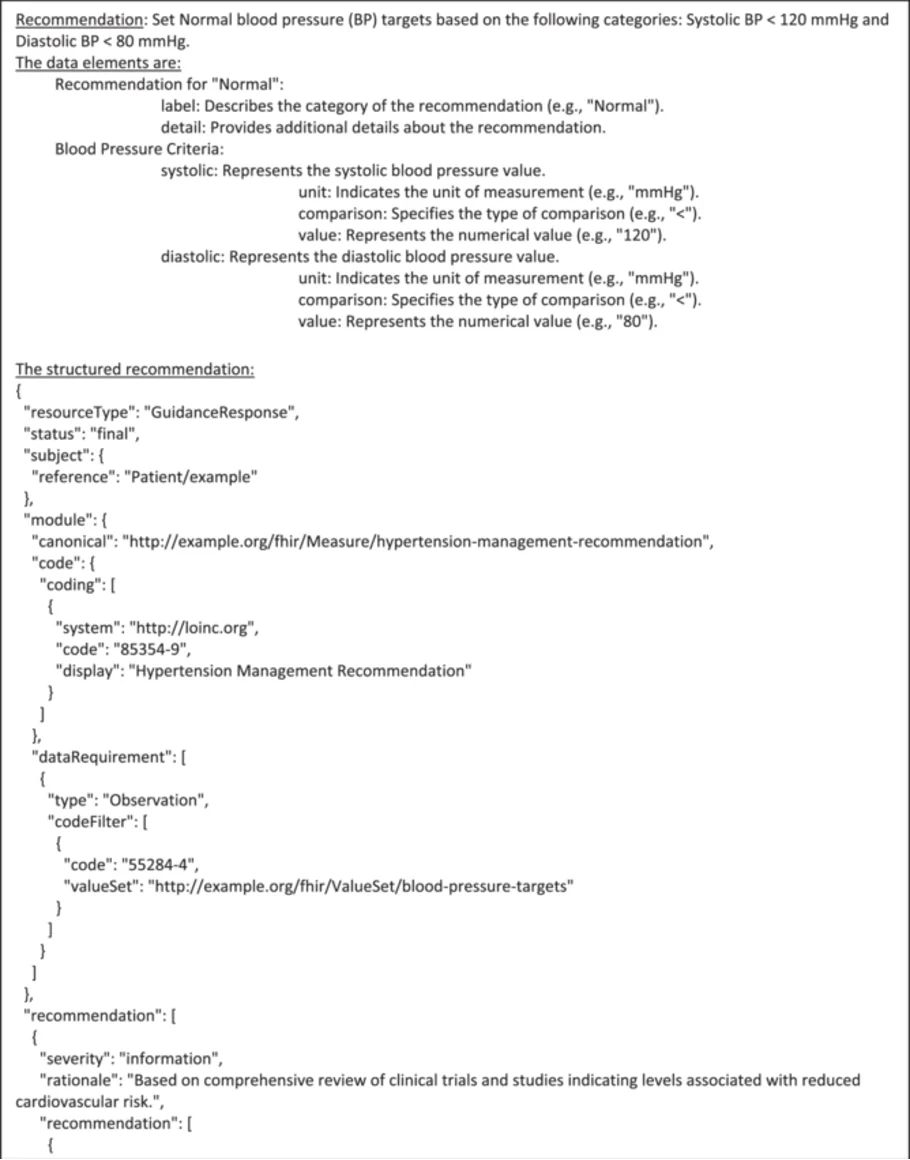
Example of data elements and value sets structured in recommendation for the management of hypertension using the CPG‐on‐FHIR® IG.^14^

**Figure 6:**
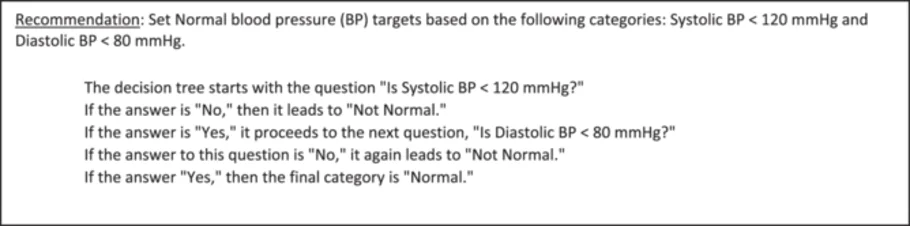
Example of decision tree approach for recommendation on the management of hypertension.

Ensure that guidance for each extracted and translated recommendation aligns with the essential data elements, value sets, and measures specified in the PICO/PECO questions, while also being expressed with unambiguous language using the CARDS methodology.^23^ Consistently employ common data elements and variables in the curation and FAIR indexing of recommendations to meet real‐world requirements, adhere to clinical terminology, and align with health outcomes.^20^, ^21^, ^22^ Data elements, such as sex, age, and identifiers, must be clearly defined and utilized throughout the LHS pertaining to the specific topic, disease, drug, screening, etc. This model aims to balance efficiency and effectiveness of decision‐making. Where possible, guideline developers should develop decision trees, often labelled as care algorithms or pathways, that support decision‐making by breaking it down into key decision points or nodes (i.e., part of the L2 knowledge level).^30^ Each node represents a critical decision or branch in the care pathway. Subject the decision tree to iterative review and validation by experts in the field to ensure that the decision tree accurately reflects the best available evidence and clinical expertise. Refer to Figures 3, 4, 5, 6 for examples illustrating the hypothetical scenario relevant to recommendation statements: If condition (x AND y OR z) is clearly observed, make a specific recommendation N. Clearly document the x, y, and z conditions statements to enhance transparency and reproducibility.

**Item 9‐Guideline Update**

(Applicable to both Guideline Developers and Informaticists/Implementers)

(New item linked to GDC topic 18 “Updating”)

Maintain open, transparent, and regularly reviewed and updated guideline recommendations based on the latest evidence available.^31^ This creates a foundation for a continuous cycle of evidence generation, translation to practice, and practice driven data collection and use.^19^, ^31^, ^32^ Use existing guidance such as the living guidelines model where recommendations are modularly updated as soon as relevant new evidence becomes available or the CheckUp tool that informs guideline developers about strategies for updating clinical guidelines and their reporting requirements.^31^, ^32^ Follow a consistently systematic approach for assigning and managing versions of all guideline updates using common version algorithms or mechanisms that ensure changes made to a file or a set of files are tracked, documented, and organized in a structured manner. Use a transparent and consistent labelling system for updates to indicate if they represent minor changes (i.e., not critical for clinical decision‐making) to major updates (i.e., recommendations are altered in the narrative and impact decision‐making). Annotating a section in the narrative that lists all changes in updated guidelines best facilitates informaticists work when computable formats need updates to be used at point‐of‐care. The modelling of versions of narrative guidelines with persistent identifiers and their linked evidence documents and supporting data is critical in evaluating reproducibility of guidance for optimal care. The guideline community may consider extending the work of the NISO Standards Committee on Versions of Journal Articles to meet the unique use cases of guideline developers.^33^ For FHIR® outputs of computable recommendations, one common approach for versioning informaticists is known as Semantic Versioning (SemVer) that provides a structured way to communicate changes in software.^34^, ^35^ SemVer allows developers and users of the software to quickly understand the nature of changes, making it easier to manage dependencies and assess the impact of updates.

## 4 DISCUSSION

This article summarizes the essential components for the expansion of the GIN‐McMaster GDC to support the development of narrative guidelines that are more readily made computable and implementable. These components were developed as part of GIN‐NA's HCD effort on “Bringing Guidelines to the Digital Age” and followed a consensus‐building process that included iterative refinement of considerations for CGs. While narrative clinical guidelines offer valuable insights, their integration into healthcare systems pose many challenges. The traditional format of guideline recommendations lacks interoperability and creates barriers to efficient incorporation into clinical workflows through digital tools such as clinical decision support systems or quality measures. The transition from narrative guidelines to actionable, computable ones is crucial for realizing the full potential of evidence‐based medicine in daily clinical practice. Therefore, enhancing the current infrastructure for authoring, publishing, indexing, and updating narrative clinical guidelines is highly needed to ensure that guidelines remain current and reflective of the latest medical knowledge while leveraging the power of cutting‐edge technology. Regular reviews and revisions can incorporate emerging evidence and adapt to evolving and innovative healthcare practices.

With an emphasis on standardization and machine‐readability, development of the L1 Checklist involves writing narrative guideline recommendations that are more easily translated into a structured format that facilitates automated processing and interpretation in healthcare systems' infrastructure and data flows. A major strength of this effort is that this extension includes an applied and clear list of recommended components for the integration of informatics and computable representation into the clinical guideline development process. This study has been validated by a team of experts representing guideline developers, care providers, patients, informaticists, and implementers. Another advantage lies in the extension format – a checklist – which is evident in its application, particularly in developing operational guidelines that would enhance interoperability, decision support, and overall efficiency in healthcare delivery. The L1 Checklist's utility has been further supported by the iterative team process to refine the checklist to provide clear guidance and support for CG development and implementation. As a possible limitation or challenge, the checklist has not been validated in terms of the feasibility to involve informatics expertise and the associated cost relative to traditional guideline development. It is expected that there may be a larger upfront cost to develop clearer narrative guidelines that can be structured and normalized according to interoperable community standards, so they are more readily made computable for implementation. However, the downstream savings in disseminating shareable, interoperable CGs instead of each clinical organization interpreting the guidelines separately and developing digital tools for implementation in an unnecessarily redundant way should result in the overall system or exchange between systems costing less.

## 5 CONCLUSION

Computable guidelines can significantly improve the quality of healthcare delivery by providing clinicians with up‐to‐date evidence‐based recommendations within clinical workflows and reducing errors and variations in care. Collaboration among guideline developers, implementers, and informaticists from the beginning of the development process is essential to successfully achieve this transformation. Integration of the components of this GDC extension within the guideline development process will facilitate the application of recommendations while reflecting the latest evidence‐based practices.

## AUTHOR CONTRIBUTIONS

**Chirine Chehab**: Conceptualization; investigation; writing—original draft; methodology; validation; visualization; writing—review and editing; software; project administration; formal analysis; data curation; supervision; resources. **Stacy Lathrop**: Conceptualization; investigation; writing—original draft; methodology; validation; visualization; writing—review and editing; formal analysis; data curation; resources. **Christopher G. Harrod**: Conceptualization; investigation; writing—original draft; methodology; validation; visualization; data curation; resources. **James Kariuki**: Conceptualization; investigation; writing—original draft; methodology; validation; visualization; data curation; resources. **Derek Ritz**: Conceptualization; investigation; writing—original draft; methodology; validation; visualization; data curation; resources. **Maria Michaels**: Conceptualization; investigation; funding acquisition; writing—original draft; methodology; visualization; validation; writing—review and editing; data curation; resources; project administration.

## CONFLICT OF INTEREST STATEMENT

The authors declare no conflict of interest.

## ETHICS STATEMENT

The authors have nothing to report.

## Supporting information

Supporting information.

## Data Availability

The data that support the findings of this study are included within the paper and in the supplementary material of this article. There are no data sets used or analyzed during the current work.
