# Supplementary material for: Development of computable guidelines: GIN McMaster guideline development checklist extension for computable guidelines
Source: Clin Public Health Guidel. 2025 May 2;2(3):e70023. doi: 10.1002/gin2.70023 (PMC12416367; doi:10.1002/gin2.70023)
Supplement: Supplementary file 1 [file GIN2-2-e70023-s001.docx]

**L1 Checklist**

This checklist is an extension of the widely used GIN-McMaster Guideline Development Checklist and Tool for the Adoption and Implementation of Computable Guidelines. This checklist extension is intended to be used as a tool to help guideline developers develop narrative clinical guidelines with knowledge Level 1 (L1) in clearer, consistent, and more structured approach so that their key information like metadata, evidence, and recommendations may be successfully implemented by informaticists in computable formats used in clinical decision support systems that would improve patient outcomes through evidence-based medical practice in a learning health system based on FAIR (Findable, Accessible, Interoperable, and Reusable) principles. This may cover clinical practice guidelines and their related systematic reviews, evidence reports, and supplementary data and materials[^1^](https://journals.lww.com/ajmqonline/fulltext/2023/09002/adapting_clinical_guidelines_for_the_digital_age_.2.aspx).

| Applicable to Guideline Developer | Applicable to Informaticist/ Implementer | Item Label | Description | Useful Resources/ Examples | Further details |
| --- | --- | --- | --- | --- | --- |
| X |  | 1. Policy | Provide a clear policy and link with a persistent identifier to public documentation of procedures used by the organization for guideline recommendations development, including how it establishes transparency, composes its guideline development group, manages conflicts of interest, reviews and rates evidence using rigorous methods, conducts peer review, updates the guidelines versions, manages data, and distributes/publishes guidelines recommendations. | - [IOM Guidance](https://drive.google.com/drive/folders/1kFhyum_ezBAHqmAYKk43ptdySCP1ruXc) - [WHO handbook for guideline development, 2nd Edition](https://www.who.int/publications/i/item/9789241548960) | New item linked to GDC topic 1 “Organization, Budget, Planning and Training” |
| X |  | 2.Scope | Clearly define the scope of the guideline development project, including who the guidelines are for (e.g., subject matter experts (SMEs), providers, patients, etc.), what clinical domains they would cover, and what settings and contexts they may be practiced at. | - [Adapting Clinical Guidelines for the Digital Age](https://journals.lww.com/ajmqonline/fulltext/2023/09002/adapting_clinical_guidelines_for_the_digital_age_.2.aspx) - [An Integrated Process for Co-Developing and Implementing Written and Computable Clinical Practice Guidelines](https://www.ncbi.nlm.nih.gov/pmc/articles/PMC10476601/) - Integrated Process tables: <https://stacks.cdc.gov/view/cdc/131006>[An Evaluation Framework for a Novel Process to Codevelop Written and Computable Guidelines](https://www.ncbi.nlm.nih.gov/pmc/articles/PMC10476596/) - Evaluation framework tool: <https://stacks.cdc.gov/view/cdc/131007> | Extended item linked to GDC topic 5 “Identifying Target Audience and Topic Selection” and topic 8 “Patient/population, Intervention, Comparison, and Outcomes (PICO) Question Generation” |
| X |  | 3.Goal(s) | Clearly articulate why a guideline development project is needed. |  | Extended item linked to GDC topic 2 “Priority Setting” |
| X | X | 4.Contributor(s) | Ensure the involvement of informaticists and/or implementers in your guideline development panel at the start of the process to help identify areas in the guideline that require clarification and translation into CG recommendations using appropriate tools, data elements and value sets. |  | Extended item linked to GDC topic 3 “Guideline Group Membership” |
|  | X | 5.Knowledge Levels Requirements | Provide a clear, strong, and tangible definition of the requirements of each guideline’s knowledge representation levels starting with the structured knowledge level 2 (L2), semi-structured knowledge level 3 (L3), and executable knowledge level 4 (L4) for guideline developers to be able to understand what is needed to write an L1 recommendation for translation by the informaticists. These levels represent a progression from traditional narrative text to fully computable formats in clinical practice guidelines development. | - [FHIR Clinical Guidelines (v1.0.0) (STU 1) (hl7.org)](https://hl7.org/fhir/uv/cpg/documentation-approach-06-01-levels-of-knowledge-representation.html#:~:text=%E2%80%9CLevels%E2%80%9D%20of%20Knowledge%20Representation%20Framing%201%20Approach%20The,Semi-structured%20...%204%20Structured%20...%205%20Executable%20) | New item to GDC topics |
| X |  | 6.Guideline Question formulation | Formulate measurable and/or observable clinical questions using the Patient/population, Intervention, Comparison, and Outcomes (PICO) or Patient/population, Exposure, Comparison, and Outcomes (PECO) approaches. | - [Patient/population, Intervention, Comparison, and Outcomes (PICO) Process](https://www.cochranelibrary.com/en/about-pico) | Extended item linked to GDC topic 8 “Patient/population, Intervention, Comparison, and Outcomes (PICO) Question Generation” |
| X | X | 7.Guideline Structure | Use a valid grading system and approach to grade CG recommendations, such as the Grading of Recommendations Assessment, Development and Evaluation (GRADE) methodology. CG recommendations need to be written and styled in an identifiable, consistent format (i.e., contained in a labelled recommendation section with each recommendation labelled so it may be provided unique identifiers) so that they may be encoded and extracted from narrative contexts for translation across interchangeable formats (i.e., conditional statements using If, then) including the strength of the recommendation (i.e., strong, or conditional). | - [JATS4R](https://jats4r.org/) - [IOM Guidance](https://drive.google.com/drive/folders/1kFhyum_ezBAHqmAYKk43ptdySCP1ruXc) - [EBMonFHIR - Clinical Decision Support - Confluence (hl7.org)](https://confluence.hl7.org/display/CDS/EBMonFHIR) - [Agency for Healthcare Research and Quality (AHRQ) Systematic Review Data Repository (SRDR+)](https://www.ahrq.gov/cpi/about/otherwebsites/srdr.ahrq.gov/index.html) | New item linked to GDC topic 12 “Judging Quality, Strength or Certainty of a Body of Evidence”.  [Use the evidence-based structure and requirements for guidelines related systematic reviews, evidence reports, and supplementary data and materials](https://docs.google.com/document/d/1sHNuICuXuiWFH1iSFLkUhea0YpDWjY33/edit?usp=drive_link&ouid=110694559203203173816&rtpof=true&sd=true). |
| X | X | 8.Documentation requirements per recommendation | Utilize existing applications, consistent templates, or authoring and conversion tools that facilitate the extraction of individual recommendation statements to be translated for execution. Such translations may be specified algorithms or decision trees or other computable representations that permit guidelines to be successfully integrated and implemented at point-of-care, allowing for systematic and consistent decision-making based on specific conditions or criteria. These resources can streamline the guideline development process and ensure a standardized approach to their documentation. Articulate all possible care options explicitly, incorporating known variables and contextual factors necessary for optimal decision-making.  Ensure that guidance for each extracted and translated recommendations aligns with the essential data elements, value sets, and measures specified in the PICO/PECO questions. Consistently employ common data elements and variables in the curation and FAIR indexing of recommendations to meet real-world requirements, adhere to clinical terminology, and align with health outcomes(21-23). Data elements, such as gender, age, and identifiers, must be clearly defined and utilized throughout the learning health system pertaining to the specific topic, disease, drug, screening, etc. This model aims to balance efficiency and accuracy in decision-making, where possible, guideline developers should provide decision trees, often labelled as care algorithms or pathways, that supports decision-making by breaking it down into key decision points or nodes. Each node represents a critical decision or branch in the care pathway. Subject the decision tree to iterative review and validation by experts in the field to ensure that the decision tree accurately reflects the best available evidence and clinical expertise. Refer to Figures 3, 4, 5, and 6 for examples illustrating the hypothetical scenario relevant to recommendation statements: If condition (x AND y OR z) is true, make a specific recommendation N. Clearly document the x, y, and z conditions statements to enhance transparency and reproducibility. | - <https://goodwin.libguides.com/c.php?g=309484&p=2066254> ] - [United States Core Data for Interoperability (USCDI) \| Interoperability Standards Advisory (ISA) (healthit.gov)](https://www.healthit.gov/isa/united-states-core-data-interoperability-uscdi) - [Value Set Authority Center (nih.gov)](https://vsac.nlm.nih.gov/) - [CDS Authoring Tool (ahrq.gov)](https://cds.ahrq.gov/authoring/documentation#CDS_Authoring_Tool_User_Guide) - [FHIR Clinical Guidelines (v0.2.0) (Current) (hl7.org)](https://hl7.org/fhir/uv/cpg/methodology-case-study.html) - FFT Decision Tree [Example](https://pubmed.ncbi.nlm.nih.gov/29484787/)   Additional Resources for International Community:   - [International Patient Summary IG (hl7.org)](https://hl7.org/fhir/uv/ips/2019Sep/) - [International Classification of Health Interventions (ICHI) (who.int)](https://www.who.int/standards/classifications/international-classification-of-health-interventions) | New item linked to GDC topic 14 “Wording of Recommendations and of Considerations of  Implementation, Feasibility and Equity” and topic 16 “Dissemination and Implementation”.  Define the data elements and variables that will be used when performing complex queries on available data sets and published literature. |
| X | X | 9.Guideline Update | Maintain an open, transparent, and regularly reviewed and updated guidelines recommendations based on the latest set of evidence available. This creates a foundation for a continuous cycle of evidence generation, translation to practice and practice driven data collection and use. Use existing guidance such as the living guidelines model where recommendations will be updated as soon as relevant new evidence becomes available or the CheckUp tool that informs guideline developers about strategies for updating clinical guidelines and their reporting requirements. Follow a consistently systematic approach for assigning and managing versions of all guideline updates using common version algorithms or mechanisms that ensure changes made to a file or a set of files are tracked, documented, and organized in a structured manner. Use a transparent and consistent labelling system for updates to indicate if they represent minor changes that are not critical for clinical decision-making to major updates where recommendations are altered in the narrative and impact decision-making. Annotating a section in the narrative that lists all changes in updated guidelines best facilitates informaticists work when computable formats need updates to be used at point-of-care. The modelling of versions of narrative guidelines with persistent identifiers and their linked evidence documents and supporting data is critical in evaluating reproducibility of guidance for optimal care. The guideline community may consider extending the work of the NISO Standards Committee on Versions of Journal Articles to meet the unique use cases of guideline developers. For FHIR outputs of computable recommendations, one common approach for versioning informaticists may consider is known as Semantic Versioning (SemVer) that provides a structured way to communicate changes in software, which thus allows developers and users to quickly understand the nature of changes, making it easier to manage dependencies and assess the impact of updates. | - <https://semver.org/> - <https://www.hl7.org/fhir/valueset-version-algorithm.html> - [Living systematic reviews: 4. Living guideline recommendations (sciencedirectassets.com)](https://pdf.sciencedirectassets.com/271297/1-s2.0-S0895435617X00127/1-s2.0-S0895435617306625/main.pdf?X-Amz-Security-Token=IQoJb3JpZ2luX2VjECcaCXVzLWVhc3QtMSJHMEUCIDyWLgusnO%2BeFo1QFslyfWsPZ29M%2Bp8X8MqmPr3AjjiIAiEA0q9xDrJLSYzayYnx6ATw89EkKd8HqGeq8OuOT39Gxx4qswUIMBAFGgwwNTkwMDM1NDY4NjUiDHwQX1dl4A%2F5Xjt0qyqQBTxvNcUkuivANXG8j1tM3LX3ehGxQPxkdStF%2BOjQ%2F3KYpxjQo6vv314iSNR9cdc2qKnH%2Fro05Bxw2I0pPEx49buekY31PFK04CHN7e4y0jszYNKN73NtzexZkrga5bwBnhUBS5Mw%2BcB3q4aGc27%2F5g8WSN5nwEmKrtskIRAogzbjbLYzC4GAD%2Bty%2BRnd7LnyFNxKCpW%2FacY9tp7sO4DcO6pM4%2BERsEZY%2Fvjrq9r52OkhqYVit54Bb%2FRlYyrCdhtXBI1qs45utnuWCy5fbf2%2Fl6t7r49jUQjxIxgTGrkciBls46bTXpIgsoFTq23lHLXt39ofn6hrpq84Rp4KNlzeJrzTkE%2FRQ6AmkFUHq4BAxwNGJbI8xfNJOyQEaj%2BDlWzyI32A%2BnXQnmClZN3vKMovutXIaO2ab5mRC20T%2BU%2BJ7SozFZG7aNQYdGJ1xVT76x%2FKM1TtR4e6V%2FKYGVqJOKuuYRdtFutGe1RvSi%2B%2BSt4oEc7q46MxXGvOksMOzeWwiSOk%2FiQtCvSAM4%2FAMnEd%2F6KhUdRb2XuHy6k6GsQ34poPFl68laZwcUdgAUoRZlUT4kiIWxza77CwXGgwe%2BzJz2ACqu2lfTOkmx63ez%2FvQ47t8UBWpmwojg5T60zZ6XDOg4P%2BT%2FKR92btnlk2JaoIhAe01FmgSngrPUje5EZgkx6aZP%2FFZoE6z6MZmWCwsFvARMj%2BhTOrN6E6aDTrcmSp7I%2B3EYHDnq6azX02mRfdtYmCB%2FoS89YSGIv9kB%2FNa8lQiKpu6rbf7cfNYCs9MIYNSDUWrhoJhMtX7PqFsJefQPz5bdhnJQaLVIpz66zt0BobfRI8k6r74W9LmdR2uZnXOJPB9ENvE%2Fkiy%2BNAL%2Fizm9x1NGxkMOervK8GOrEBzWZ%2BIfCojST1dxeJkNNmcOPlbW2EkyKwFxXz5LQ5PXDVbvNdPQmTVec3rGeL36soTiUfaoZ%2FuFNqeGCDH8BXssl4Og9XNuu%2B%2FC2MJCt5AK06LoHbneW4jLW93g6xlWiTnEd6LwwopXXK3f3pPEoga6ZTdeUy%2BL1QN3jzn4Xl3wYniUdvx0j1TFuo8lucwxAifQVMr99CKaIm3wkNoMqp2%2FbHlQujgO2TCF%2BNKJKQuYZl&X-Amz-Algorithm=AWS4-HMAC-SHA256&X-Amz-Date=20240311T151918Z&X-Amz-SignedHeaders=host&X-Amz-Expires=300&X-Amz-Credential=ASIAQ3PHCVTYRMAAG2VZ%2F20240311%2Fus-east-1%2Fs3%2Faws4_request&X-Amz-Signature=acb1a9bdcde6b09a0d3f075455f20c4a55a15c95d607dac0f78c2e3d3ac534b7&hash=e033f92c092f838478775ede44a5803436d77be6d93cb19f8098ef51a2236e87&host=68042c943591013ac2b2430a89b270f6af2c76d8dfd086a07176afe7c76c2c61&pii=S0895435617306625&tid=spdf-72408da6-5fd2-4f06-a3e5-19a384bd4e45&sid=00b2029a7612594fed5a75b-c26c30b1e0dfgxrqa&type=client&tsoh=d3d3LnNjaWVuY2VkaXJlY3QuY29) - [Reporting Items for Updated Clinical Guidelines: Checklist for the Reporting of Updated Guidelines (CheckUp) (nih.gov)](https://www.ncbi.nlm.nih.gov/pmc/articles/PMC5224740/pdf/pmed.1002207.pdf) - <https://www.niso.org/publications/z3996-2021-jats> - <https://www.niso.org/standards-committees/journal-article-versions> | New item linked to GDC topic 18 “Updating”.  Each version shall have a persistent identifier for transparency in the historical record, and that each version can be linked across all other versions of that document through clearly communicated related publications in each document and its indexed metadata. |
